# Pulse rate variability: a new biomarker, not a surrogate for heart rate variability

**DOI:** 10.1186/s40101-020-00233-x

**Published:** 2020-08-18

**Authors:** Emi Yuda, Muneichi Shibata, Yuki Ogata, Norihiro Ueda, Tomoyuki Yambe, Makoto Yoshizawa, Junichiro Hayano

**Affiliations:** 1 Department of Electrical Engineering, Graduate School of Engineering, Tohoku University, Sendai, Japan; 2 Cardiology, Mackay Base Hospital, Mackay, Australia; 3 Makabe Hospital, Higashi Matsushima, Miyagi Japan; 4 Nagoya City University Graduate School of Medical Sciences, Kawasumi 1 Mizuho-cho Mizuho-ku, Nagoya, 467-8602 Japan; 5 Institute of Development, Aging and Cancer, Tohoku University, Sendai, Japan; 6 Research Division on Advanced Information Technology, Cyberscience Center, Tohoku University, Sendai, Japan

**Keywords:** Pulse wave, Photoplethysmography, Heart rate, Heart rate variability

## Abstract

With the popularization of pulse wave signals by the spread of wearable watch devices incorporating photoplethysmography (PPG) sensors, many studies are reporting the accuracy of pulse rate variability (PRV) as a surrogate of heart rate variability (HRV). However, the authors are concerned about their research paradigm based on the assumption that PRV is a biomarker that reflects the same biological properties as HRV. Because PPG pulse wave and ECG R wave both reflect the periodic beating of the heart, pulse rate and heart rate should be equal, but it does not guarantee that the respective variabilities are also the same. The process from ECG R wave to PPG pulse wave involves several transformation steps of physical properties, such as those of electromechanical coupling and conversions from force to volume, volume to pressure, pressure impulse to wave, pressure wave to volume, and volume to light intensity. In fact, there is concreate evidence that shows discrepancy between PRV and HRV, such as that demonstrating the presence of PRV in the absence of HRV, differences in PRV with measurement sites, and differing effects of body posture and exercise between them. Our observations in adult patients with an implanted cardiac pacemaker also indicate that fluctuations in R-R intervals, pulse transit time, and pulse intervals are modulated differently by autonomic functions, respiration, and other factors. The authors suggest that it is more appropriate to recognize PRV as a different biomarker than HRV. Although HRV is a major determinant of PRV, PRV is caused by many other sources of variability, which could contain useful biomedical information that is neither error nor noise.

## Main text

Recently, many studies have reported the accuracy of pulse rate variability (PRV) as a surrogate of heart rate variability (HRV) [1–4]. Since photoplethysmography (PPG) sensors are almost universally incorporated into the widespread wearable watch devices, the pulse wave has become the most popular biosignal available in daily life, replacing electrocardiogram (ECG). Therefore, it may be natural to consider the use of PRV to assess autonomic functions and disease prognosis in the same way as HRV. However, the authors are concerned about the research paradigm based on the assumption that PRV is a biomarker that reflects the same biological properties as HRV.

This assumption could undermine the physiological and pathological value of PRV. Both PPG pulse wave and ECG R wave reflect the periodic beating of the heart. Thus, without cardiac electromechanical dissociation, pulse rate and heart rate should be equal, but it does not guarantee that the respective variabilities are also the same. As shown in Table 1, the process beginning with ECG R wave and ending with PPG pulse wave involves several steps of the transformation of physical properties. First, ECG R wave is electric potential caused by depolarization of ventricular myocytes, which triggers the contraction of the myocardium (electromechanical coupling). Second, ventricular wall contraction increases left ventricular pressure (force-pressure conversion), which opens the aortic valve when it exceeds the diastolic aortic pressure (the time up to this step is pre-ejection period). Third, the left ventricle propels the volume of blood (corresponding to stroke volume) during the opening of the aortic valve, which causes pressure impulse according to its compliance in the aorta (volume-pressure conversion). Forth, the pressure impulse generates a pulse wave, which conducts through the arterial wall at a velocity determined by the arterial radius, wall thickness, and elasticity and blood density (impulse-wave conversion). Fifth, the pulse wave reached the site of PPG measurement in pulse conduction time and increases blood volume in the tissue vascular bed (pressure-volume conversion). Finally, the changes in blood volume cause changes in reflected/transmitted light intensity, which are detected by the PPG sensors (volume-light intensity conversion). Each of these six steps has a respective transfer function with different delays and frequency characteristics, and various factors, such as autonomic activities, respiration, blood pressure, and diseases, could modulate those functions directly and indirectly and also generate the intrinsic components of PRV.

**Table 1:** Factors affecting the information transmission from ECG R wave to PPG pulse wave

Physiological measure	Anatomical location	Conversion of physical property	Modulators	
			Direct	Indirect
**ECG R wave** **↓**	Left ventricular muscle	Electric excitation **↓**	Intraventricular conduction, ventricular activation time, electromechanical coupling	Myocardial ischemia, heart diseases
**Pre-ejection period** **↓**	Left ventricle	Muscle force **↓**	Preload and after-load, contractility, aortic diastolic pressure	Respiration, blood pressure, body position and exercise, heart failure (alternating pulse)
**Aortic pressure elevation** **↓**	Aorta	Pressure impulse **↓**	Stroke volume, aortic dynamic compliance, intrathoracic pressure	Respiration, peripheral resistance
**Pulse conduction time** **↓**	Artery	Pressure wave **↓**	Internal radius, wall thickness and elasticity, blood density	Vasomotor sympathetic activity, endothelial function, blood pressure
**Tissue volume** **↓**	Tissue microvasculature	Blood volume **↓**	Vascular dynamic compliance, blood flow, venous pressure	Location and body position, body and environmental temperature
**PPG pulse wave**	Red cell hemoglobin	Light intensity	Absorption, scattering, reflection, and transmission; vascular bed volume	Local red cell count, hemoglobin content, waveform fiducial point

*ECG* electrocardiography, *PPG* Photoplethysmography

In fact, there is concrete evidence of the dissociation between PRV and HRV. Constant et al. [5] reported the presence of PRV in the absence of HRV in pediatric patients with a fixed-rate implanted pacemaker. Wong et al. [6] observed differences not only between PRV and HRV but also between PRV measured from the fingers of the right and left hands. We also observed the differences in PRV measured on the forearm and wrist even in the same arm [7]. Additionally, differing effects of body posture and exercise between PRV and HRV [8] and the variance of PRV caused by the effects of differing waveform on pulse wave fiducial point have been reported [9, 10].

**Fig. 1:**
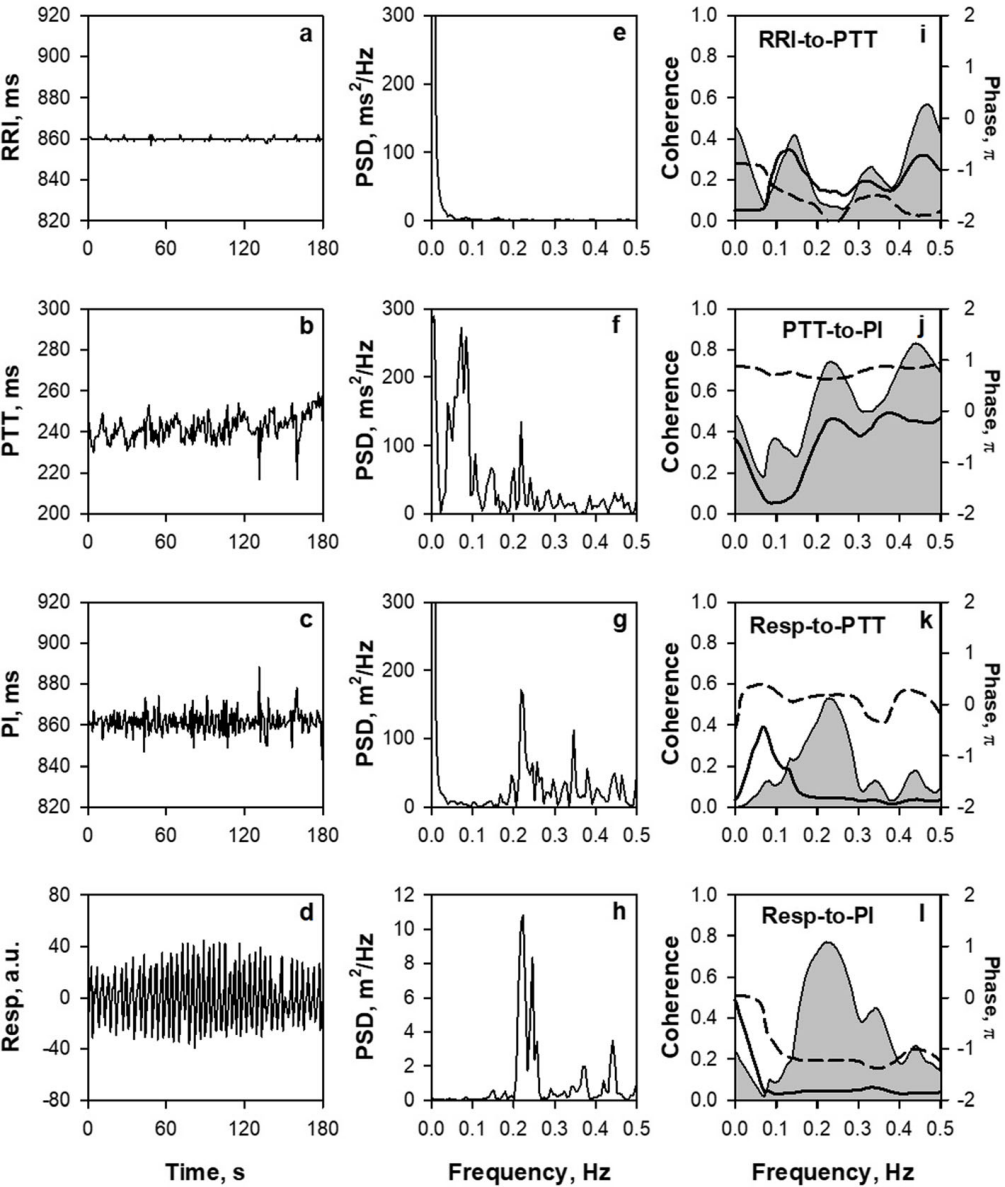
Spectrum and cross-spectrum analyses of R-R interval (RRI), pulse transit time (PTT), pulse interval (PI), and respiration (Resp) measured from simultaneous recordings of ECG, finger-tip photoplethysmography (PPG), and nose-tip thermistor respiration in a female patient (91 year) with an implanted cardiac pacemaker with a fixed pacing rate (70 bpm). All signals were recorded at 1000 Hz. PTT was measured as time from ECG R wave to PPG presystolic foot point of each beat and PI as the interval between the foot points of consecutive beats. In panels **i**–**l**, shadowed area, solid line, and dashed line represent coherence, transfer magnitude, and phase, respectively. PSD, power spectral density

Following a pioneering work by Constant et al. [5] in children, we recorded PPG simultaneously with ECG in an elderly female patient with fixed-rate ventricular pacing (Fig. 1). Despite the absent HRV and its flat power spectrum (panels a and e), fluctuations were observed in the pulse transit time (PTT) measured as the time from the ECG R wave to the PPG presystolic foot point (pre-ejection period plus pulse conduction time) (panel b). Additionally, the power spectrum of PTT had two major peaks at 0.07 and 0.22 Hz, corresponding to low-frequency (LF) and high-frequency (HF) components, respectively (panel f). This suggests that any or all of the steps comprising PTT are subject to autonomic, respiratory, and other modulations. Pulse interval also showed fluctuations, i.e., PRV (panel c), but its power spectrum had only HF peak but not LF (panel g). The absent LF in PRV is reflected in lower coherence and transfer magnitude for the LF band of the transfer function from PTT to pulse interval (panel j). Theoretically, when pulse signals with a constant interval at 860 ms are transmitted with a transit time that oscillates at 0.07 Hz, pulse signals observed at the transmission destination show interval variations with a power of 0.14 times the transit time oscillation. Conversely, when the transit time oscillates at 0.22 Hz, the power of the observed pulse signal interval variation is amplified 1.25 times, and when it oscillates at 0.3 Hz, the power is amplified 2.0 times the transit time oscillation. This indicates that the higher the PTT fluctuation frequency, the more amplified power the PRV is generated. This suggests that the mechanisms of increased PRV HF power during standing and exercise [3, 8] may be attributable at least partly to an increase in respiratory frequency. These phenomena indicate that fluctuations could be generated and modulated by autonomic functions, respiration, and other factors differently in R-R intervals, PTT, and pulse intervals, suggesting that PRV is a signal containing the physiological and pathological information that is not contained in HRV.

## Conclusions

The authors suggest that it is more appropriate to recognize PRV as a biomarker different from HRV. Although HRV is a major source of PRV, PRV is generated and modulated by many other sources and factors, which could contain useful biomedical information that is neither error nor noise. From this aspect, there seem more valuable approaches than pursuing the accuracy of PRV as a surrogate for HRV. One is to analyze HRV and PRV simultaneously to find physiological and clinical values of their differences in time and frequency domains and nonlinear dynamics, i.e., the noninvasive analyses of dynamic conduction properties of impulse train generated by the heart. The other is to directly investigate the biomedical usefulness of PRV itself, independent of HRV.

## Data Availability

The datasets used for the current study are available from the corresponding author on reasonable request.

## Abbreviations

ECG: Electrocardiogram
HRV: Heart rate variability
PPG: Photoplethysmography
PRV: Pulse rate variability
